# Improving Situation Awareness via a Situation Model-Based Intersection of IoT Sensor and Social Media Information Spaces

**DOI:** 10.3390/s22207823

**Published:** 2022-10-14

**Authors:** Irfan Baig Mirza, Dimitrios Georgakopoulos, Ali Yavari

**Affiliations:** School of Science, Computing and Engineering Technologies, Swinburne University of Technology, Melbourne, VIC 3122, Australia

**Keywords:** situation awareness, IoT, social media, situation modelling, information fusion

## Abstract

Existing techniques for distilling situation awareness currently focus on information harvested from either IoT sensors or social media. While the benefits of fusing information from these two distinct information spaces for achieving enhanced situation awareness are well understood, existing techniques and related systems for fusing the IoT sensors and social media information spaces are currently embryonic. Key challenges in intersecting, combining, and fusing these information spaces to distil high-value situation awareness include devising situation models and related techniques for filtering, integrating, and fusing sparse and heterogeneous IoT sensor data and social media postings to provide a richer and more accurate situation awareness. This paper proposes novel, semantically based techniques fusing social media and IoT sensor information spaces and a comprehensive, fully implemented system that utilizes these to provide enhanced situation awareness. More specifically, this paper proposes the design of semantic-based situation models for fusing sensor and social media information spaces and presents techniques for finding similarities across these information spaces and fusing social media posting and IoT sensor data to generate an enhanced situation awareness. Furthermore, the paper presents the design and implementation of a complete system that uses the proposed models and techniques and uses that in an experimental evaluation that illustrates improvements in situation awareness from fusing the IoT sensor and social media information spaces.

## 1. Introduction

We are living in a world of billions of Internet of Things (IoT) sensors and Social Media Networks (SSNs) [1,2] that are constantly providing us with an enormous amount of information that is critical for situation awareness of the physical world. Widely used IoT and SSNs technologies allow machines (e.g., sensors, vehicles, and industrial machines) and people to create digital information (which we refer to as IoT sensors and Social Media information spaces, respectively) from observations of the physical word and communicate that via the Internet. More specifically, IoT sensors are machines that automatically monitor the physical world, while human or social sensors are people that report their observations of situations and thoughts via postings on social media such as Twitter [3,4,5,6,7,8,9,10,11], Facebook [11], Reddit [12], Weibo [13,14,15], and TripAdvisor [16]. Today IoT and social media data represent a significant (if not the dominant) portion of the volume of Internet data traffic and offer a great opportunity to increase the scope and accuracy of situation awareness that is needed to understand and respond to virtually any situation.

Situation Awareness is a concept that was initially used in military services and situation awareness, the perception of elements in the environment within a volume of time and space, the comprehension of their meaning, and the projection of their status in the near future [1,2,17,18]. While perception is being aware of the elements such as the sensor measurements and social media respect to the decision maker’s goals. These elements when put together help the decision maker form a holistic picture of the environment. In this paper we aim to improve the comprehension by the mapping, translation, and fusion of high value information from sensors and/or social media.

Traditional situation awareness solutions consider information spaces from either IoT sensors or social sensors, and the full potential of combining these information spaces is still far from being fully realized [6,7,8,10,13,14,15,19,20,21,22]. This is due to the lack of any solution that will provide an efficient intersection of high value information from the combined information space. In this paper we contribute to the development of a novel approach for combining the social media and IoT sensor information spaces for providing improved situation awareness. This is accomplished by devising and using situation models for describing situations of interest, and techniques that utilize these models to fuse related data in these information spaces to provide more comprehensive and accurate situation awareness information. To illustrate the improved situation awareness from the combined information spaces, the paper uses examples from wind gust-related situations. We developed a situation model for describing wind gust-related situations in both IoT sensor and social information spaces. To assess situation awareness improvement, we designed and implemented a novel system for intersecting sensor and social information spaces. This system is highly scalable, utilizes distributed cloud computing services and performs near-real time data processing across all information spaces. This paper includes the following novel contributions:

- A situation model that captures weather-related situations that involve both IoT sensors and social media information;
- Techniques for mapping and fusing social media postings and IoT sensor data observations to the situation model;
- The design and implementation of a complete system that performs the above;
- An experimental evaluation that shows the benefits of intersecting high value information from IoT sensors and social media information spaces for improving situation awareness.

The rest of this paper is organized as follows: Section 2 presents the related work in situation awareness not using a situation model, situation awareness focused in the IoT sensor information space, situation awareness in the social media information space, and related work in the combined information space. Section 3 presents a scenario illustrating the benefits of intersecting sensor and social media data to improve situation awareness for wind gust-related situations. Section 4 presents the semantic situation modelling. Section 5 discusses the mapping of sensor data to a semantic situation model. Section 6 discusses the mapping of social media data to a semantic situation model. Section 7 discusses combining and intersecting social media data and sensor data into a semantic situation model for improved situation awareness. Section 8 discusses the evaluation of intersecting the information spaces of sensors data and social media postings. Section 9 concludes the paper and describes the potential future research directions.

## 2. Related Work

In the following sections we present related work from the following perspectives: situation awareness not using a situation model for this (Section 2.1), situation awareness focused in the IoT sensor information space (Section 2.2), situation awareness in the social media information space (Section 2.3), and situation awareness in the combined information space (Section 2.4).

### 2.1. Situation Awareness Not Using Situation Modelling

To explain the related work, we need to first discuss what is the contribution of the situation model for improving situation awareness to the following: (a) describing situations and (b) intersecting information from combined information spaces using the concepts from the situation model. For that we consider wind gust-related situations in the state of Victoria as an application described in Section 3 to explain this and also to present a high-level overview of the proposed approach.

A significant relationship has been observed between the frequency of the tweets to that of the corresponding situations [10]. However, using the frequency of selected social media postings as a feature translation parameter might not reflect the actual situation. For example, in a situation awareness of the most recent rainfalls in the state of Victoria in June 2021, social media postings such as (1) “*Recent heavy rains resulted in back-flow from Lake Victoria, damaging homes in the area*” and (2) “*Victoria has been hit particularly hard in the last 18 months. Fire, plague, flood*” might not reflect the actual situation as the actual discussion might be referring to another location or a situation in the past as the first social media posting is referring to the Lake Victoria in Africa. Moreover, with frequency-based feature translation, the frequency of social media content depends on the social media adoption (active users) in the affected area. Moreover, social media activity is generally associated with the geographical and demographical distribution of users; the social and spatial heterogeneity in the usage of social media services might bring some biases for the analysis of the actual situation [13]. Moreover, situations in the real world have a more complex structure which includes relations between entities, the spatio-temporal aspects, and often requires additional semantic interpretation to fully understand the situation. A situation model models these aspects when describing situations and supports the extraction of high value information that can be used for a complete situation awareness.

### 2.2. Situation Awareness in Sensor Information Spaces

In this section we discuss the current state of the art in the use of IoT sensor information spaces for situation awareness. Situations can be further classified as simple and complex. Simple situations involve only space, time, or a single basic concept or basic data type (e.g., a keyword in social media, a value of a simple data type such as temperature). Complex situations need to be modelled to be understandable, i.e., they can be described using a model involving features from IoT sensors and social sensors. In the IoT sensor information space, various techniques have been used for situation awareness. Techniques such as deep learning have been applied to achieve situation awareness in various applications such as cloud-assisted IoT for industrial control systems [23] and Smart Health [24]. The authors of [23] proposed two deep learning-based adaptive threat detection models. The first model uses a disjoint training and testing data to build a deep belief network (DBN) and a corresponding Artificial Neural Network (ANN). DBN models do not require additional processing in feature reduction and unsupervised clustering before being trained for supervised learning. The second model further trains the DBN on unlabeled data to provide additional knowledge on changes in malicious attack patterns. Convolutional neural networks (CNN) [24] have been applied to fuse information from stereoscopic cameras and infrared sensors to identify ecologically valid obstacles when using Electronic Travel Aids. A detection layer maps convolutions to a fixed feature vector, and a fully connected network takes the fixed feature vector as the input and outputs a class label and bounding box coordinates. Objects, i.e., features of interest were identified based on the class labels or as general obstacles. Feature upsampling was applied to boost the detection of small objects without significantly increasing time and complexity.

Several ontologies have been used for situation awareness using IoT sensors. SSN (created by W3C) is a generic ontology mainly related to a sensor and its observations. It is an ontology developed by the Semantic Sensor Network Incubator group for describing sensors, the procedures involved, their features of interest, the sample strategies, observed properties, and actuators. SOSA —Sensor observation, Sample, Actuator—developed by the first joint working group of the Open Geospatial Consortium (OGC), provides a formal lightweight general-purpose specification for modelling entity interactions. SOSA, while providing a lightweight core for SSN [25], defines common classes and properties for safe data exchange and also acts as a replacement for SSN’s Stimulus Sensor (SSO) core. SOSA [26] provides an event-centric perspective and is centered around observations, sampling, actuations, and observation procedures. The Smart Appliances REFerence (SAREF) [27] ontology evolved from the needs of smart home solutions such as smart ovens and refrigerators.

Situation models have been reused, adapted, or modelled in many ways in the literature using ontologies to effectively describe the situations. Su et al. [28] modelled every observation as an RDF statement. RDF is a conceptual graph-based data model commonly used to represent arbitrary structures, where a graph consists of statements with (subject, predicate, object) structure. This structure can be interpreted as: “object o stands in relationship *p* with subject s” [28]. Serialization formats for RDF include RDF/XML, RDFa, N Quads, Notation 3 (N3) N-Triples, Turtle, JSON for Linked Data (JSON-LD), and Entity Notation (EN), etc. [28]. RDF Schema (RDFS) provides a vocabulary to describe application-specific classes and properties whereas OWL provides a more complex vocabulary which can be helpful for modelling complex ontologies [28]. Web Ontology Language (OWL) [28,29,30,31] based on the W3C web standard is preferred over the more verbose RDFS/XML because of its expressiveness and reasoning ability [31].

Most of the existing works model features of interest as classes [29,31] and their respective features as subclasses while leveraging [29,31] the properties to describe the internal relationship between classes. The situation model of Hussein et al. [29] included classes and subclasses in them SIoT ontology (domain-specific ontology for smart spaces) to model features from the physical world, i.e., location, objects, etc., as well as features related to the users and their profiles (including their preferences). In their model, properties are leveraged to model detailed aspects about features such as *hasTemperature*, *hasLatitude*, and *hasHumiditylevel* which may be used to capture the values of temperature, latitude, and humidity level, respectively. Their model aimed at representing physical things in addition to social knowledge about users and smart services.

A microservice-based situation model was used to understand situations in cross-domain IoT applications by modelling features as virtual objects and composite virtual objects [30]. The virtual objects virtualize and provide information about real-world objects. Another situation model included processing natural language to detect home appliances based on an ontology that describes home appliances and identifies the action to be executed by determining the command from the natural language. The observational model in home appliances ontology [32] describes, among other things, the states, sensors, actions, alerts, and services that a home appliance provides and also uses these descriptions to recognize and classify entities. This model also classifies the instruction provided in the natural language to be an executional command or just a query of the state of the appliance. However, the regular pattern matching rules were extended to include concepts from the home appliance ontology which were then used to identify the home appliance from the natural language query. However, this approach makes it difficult to identify instructions when the users’ instructions do not contain words (keywords) describing the home appliance or the action but contain references to them. The situation model of a network security situation described features of interest as classes, the features as subclasses, and object properties which were leveraged to describe the internal relationship among the features of interest to reflect the network security situation.

### 2.3. Situation Awareness Using the Social Information Space

In this section we discuss the techniques used for situation awareness in social media information spaces. In the literature, social sensors are commonly referred to as people posting their observations on social media such as Twitter, Facebook, Weibo, etc. Information spaces constructed using social sensors have used geolocated Twitter data to understand social distancing situations. Xu et al. [33] used a twitter mobility index, a metric based on the standard deviation of a user’s geolocated tweet as high-value information in the information space for situation awareness.

Word frequencies, bursty words [34], and NER-based rules [9] are commonly used in the literature for extracting high value information from social media information spaces. Fang et al. [13] used word frequencies to observe the temporal evolution of disaster-related topics, assess disaster impact, and identify disaster hotspots. Word frequencies [13] were used to analyze temporal variations during the evolution of the 2016 Wuhan rainstorm, and identify sub-events triggered by a hurricane situation. To identify trapped situations Rexiline et al. [9] formed a set of rules based on NER such as “person name followed by a predefined unigram, location name followed by a preposition and then by a unigram”.

Techniques such as logistic regression, Maximum Entropy Principle [7], transfer learning [12], naïve Bayes classifiers [35], CNN-based [3,36], ANN [14], and support vector machines [3,9] have been used to understand situations using social media information spaces. The maximum entropy principle has been considered for density estimation and is an effective model in the presence of a limited number of positive labels and a substantial number of features [7]. Transfer Learning approaches have been applied to understand stressful situations and classified stress expressions in Reddit posts into binary labels (high stress and low stress) [12]. A semantically enhanced wide and deep Convolutional Neural Network (Sem-CNN) model has been used to automatically detect (identity) and classify crisis information. Korolov et al. [37] used logistic regression to establish a correlation between mobilization-related social media messaging and protest occurrences. Khalifa et al. [38] used the DBSCAN algorithm to identify tweet clusters and identify outliers using outlier detection techniques.

In the presence of class imbalance problems which are commonly associated with social media data, the Naïve Bayes classifier is not suitable for classification [35]. CNN using Fasttext for word embeddings was observed to be the best performing model for health classification tasks [3]. Rexiline et al. observed that a combination of rules, linguistic features, and linear SVM improved the performance of text classification system [9]. Support Vector Machines (SVM) are supervised machine learning algorithms used for classification and regression and are more suitable for text classification than other machine learning-based algorithms because of their automatic parameter tuning property [9]. A kernel is the core of a learning algorithm and works based on a similarity function.

### 2.4. Situation Awareness in Combined Information Spaces

In this section we discuss the techniques used for situation awareness in combined information spaces, i.e., using both IoT sensors and social sensor information spaces for situation awareness, henceforth referred to as combined information spaces. Traditional situation awareness systems consider information spaces constructed either using IoT or social sensors features for situation awareness. However, more recently, combined information spaces have been constructed and used for situation awareness for several reasons discussed in Section 4. There are many situations such as flood transportation, smog, rainfall, etc., in which data from IoT sensors cannot be fully exploited [28,39] as data from rain gauges, radar, or meteorological satellites are not always available for various reasons, such as the failure of rain gauges, lack of maintenance leading to incorrect data, etc., and hence a complete awareness of situations cannot be achieved. Researchers [12,13,14,28,29,30] have identified the need for supplementing IoT sensor information spaces with social sensor information spaces for a better awareness of situations. However, most of the existing work [6,8,10,13,14,19,20] involving combined information spaces has translated social sensor features using frequency-based transformation techniques to homogenize the features so that it they are suitable for fusion. For algorithms based on machine learning, each data item must be accurately represented as a feature vector. Frequency-based transformation involves using the frequency of social media postings as a metric to achieve homogenous features. Such a transformation assumes that the intensity of the situation is directly proportional to the (activity) number of relevant social media postings [10]. For example, Fang et al. [13], Restrepo-Estrada et al. [10], Chen et al. [14], and Wu et al. [6] translated features based on social media activity.

Combined information spaces have recently been used for various reasons such as identifying the utility of IoT sensor features [13], augmenting the low temporal resolution of satellite imagery [21], and for awareness of other situations such as floods. In [10], the combined information space included features from social sensors estimated by extracting and converting Twitter messages into rainfall values based on the frequency of geo-located tweets containing flood-related keywords obtained for cumulative periods (20, 30, 40 min). Cervone et al. [20] used high-value information from social media to complement IoT sensor information spaces containing high-value information from remote sensing imagery for awareness on flood situations. A decision tree induction classifier helped in crafting high-value information based on the classification of flood-related images. The authors of [6] spatially correlated the severity of damage in a given area with the disaster-related activity reported by social sensors. A spatial correlation of features describing economic losses, geolocation, and social media activity was used to identify damage severity. Within the combined information spaces, Wang et al. [7] used high value information from social sensors to overcome noisy and spatially biased label issues in the IoT sensor information space. The geotagged information was extracted from tweets and was used as labels to complement high-value information describing satellite imagery data for flood extent estimation using spatial density estimation techniques.

## 3. Scenario Illustrating the Benefits of Intersecting Sensor and Social Media Data to Improve Situation Awareness for Wind Gust-Related Situations

A sudden increase in wind speed even for a few seconds is commonly referred to as a wind gust which can be dangerous and destructive. The Beaufort wind scale developed by Sir Francis Beaufort in 1805 is widely used by the Bureau of Meteorology (BOM)in Australia to measure wind speed based on its impact on land and sea [1]. Winds commonly feature in the Bureau of Meteorology’s weather forecast and warnings in Australia and these forecasts are made up of wind speed and wind directions which are measured in intervals of 10 min and at a height of 10 m above sea level. BOM classifies wind gust-related situations as gales, storms, or hurricanes, etc., based on the wind warning thresholds applied to wind speeds. These situations commonly cause large branches to sway and break off trees, dislodge roofs, and uproot trees while also causing significant structural damage to building infrastructure and also result in a significant loss of human and animal life. In addition to wind speed, the severity of damage is also dependent on the wind direction and duration. Significant changes in wind duration and direction have been found to contribute to a wind gust-related situation. For example, in September 2019, in Grand Bahama, hurricane Dorian [2] stalled for more than 24 h with a maximum sustained wind speed of 185 kmph and caused significant flooding and damaged numerous homes. While naturally occurring wind gust-related situations such as gales, storms, and hurricanes can be forecast, a better awareness of the situation can help in improving preparedness—making decisions on where to target the rescue efforts, minimize the consequences, and reduce the impact to vulnerable areas.

**Figure 1:**
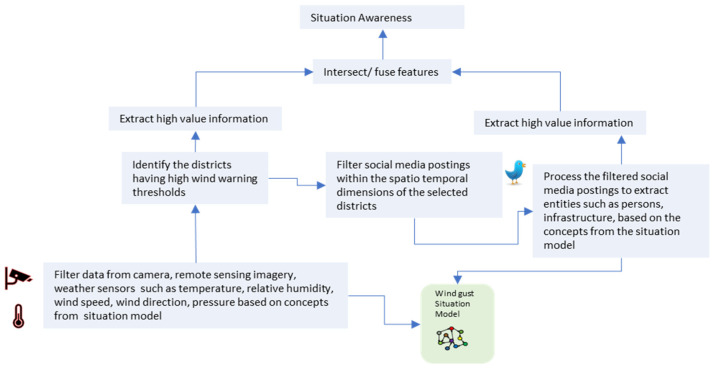
Illustration of data analysis tasks for intersecting sensor and social media data to improve situation awareness.

Various IoT-based atmospheric sensors such as temperature, relative humidity, wind speed, and wind direction, pressure, etc., can be used for the early forecasting of these situations. The data from social media postings and the images and videos contained therein, etc., can potentially be utilized to improve the awareness of these situations. Consider the Bureau of Meteorology monitoring the state of Victoria with the following IoT infrastructure: (1) IoT devices that include (a) weather sensors at weather stations including temperature, relative humidity, wind speed, wind direction, pressure and (b) satellite imagery; (2) social media postings when people post about infrastructure and utilities, caution, and advice, affected people, help requests, relief, fundraising, etc. While this information comes from IoT-based sensors at weather stations, satellite images can help us identify potential vulnerable areas:, (a) a lack of sensor coverage in the region of storms and hurricanes, etc., due to various reasons such as unavailability, satellite revisit times, etc., further complicates the awareness of a situation as it unfolds and (b) in the event of these situations, sensors cannot report first-hand information of the situation to help with situation management. The supplementary information from social media can be used to compensate for the information sparseness from sensors in improving our awareness of the situation and in the event of emergencies can also help in improving the monitoring and coordinating of rescue efforts by providing first-hand information of a situation. Figure 1 illustrates the data analysis tasks to provide situation awareness in the event of a wind gust-related situation.

Now consider a situation awareness application for identifying a potential wind gust-related situation and improving emergency management. This application should utilize the data from the IoT devices and perform the following tasks to provide an awareness of the vulnerable areas, human and animal life, injuries and infrastructure, etc.: (1) utilize a situation model that describes a wind gust-related situation; (2) based on the situation model, perform an initial analysis of weather conditions by analyzing data from weather sensors including temperature, relative humidity, wind speed, wind direction, pressure; (3) identify the districts with high wind warning thresholds as described by the situation model and provide high value information such as time, location and situation type, etc.; (4) filter social media postings within the spatio-temporal dimensions of the selected districts; (5) process the filtered social media postings to extract wind gust-related observations and based on the situation model provide high value information such as people, infrastructure affected, location, etc.; (6) fuse the high value information from sensors and social media to provide an improved situation awareness in terms of information on people who need help, disruption information such as roads, water, breaks, and flooded areas etc.

## 4. Semantic Situation Modelling

We define a *situation* as the collection of all the features, their relationships, (e.g., those that relate to ourselves, anything that we care for or are interested in), and the values of these that are critical for situation awareness. Please note that we did not aim to devise a comprehensive sematic situation modelling ontology from scratch, but to rather reuse and combine concepts from existing ontologies that are currently used for model sensor or social media information from the perspective of providing situation awareness by intersecting sensor and social media information spaces. This is discussed further in Section 8. In situation modelling, a *feature* is a specific, observable, and measurable property (characteristic) such as height, color, etc., and can be used to describe an entity such as a tree, car, person, etc. The feature values can either be a result of an observation, estimation, or calculation, etc., from IoT sensors or social sensors. Situations could involve only simple features, such as only space or time, or a basic concept such as temperature. An *observation* relates to the process of determining the value of a feature that can be sensed directly or indirectly from an environment by an IoT sensor or reported by a posting from a social sensor. So, an information space is characterized by features and this needs to be identified, searched, selected, or extracted from an IoT sensor or a social sensor to provide effective support for achieving situation awareness.In this paper we propose using ontology-based situation models for bridging the sensor and social media information spaces. We only used those classes which are just enough to describe a situation. Ontologies have been used for describing IoT sensors and their data and can model, e.g., what is being observed by capturing formal descriptions of their features and relationships known. Gruber [40] defined ontologies as a “formal explicit specification of a shared conceptualization”. By formal explicit specification we mean the ontology must be machine-readable and understandable. Shared implies the community consensus towards the ontology and conceptualization refers to the concepts and properties used to represent knowledge in a specific situation. While conceptualization represents an abstract view of the world, specification aims to provide a concrete structure to conceptualization using a standard vocabulary and semantics. A situation model is an ontology or a fragment of an ontology that possibly includes classes, subclasses, properties, etc., that are necessary to provide a formal description of the features of a situation of interest. An important characteristic of the situation model lies in its ability to describe a situation from heterogenous sensors and capture their relationships. Unlike traditional domain ontologies, situation models tend to be more compact and require less effort and time to be developed. The situation model we propose in this paper extends the standard W3C SSN ontology, and the namespaces which are listed in Table 1.

**Figure 2:**
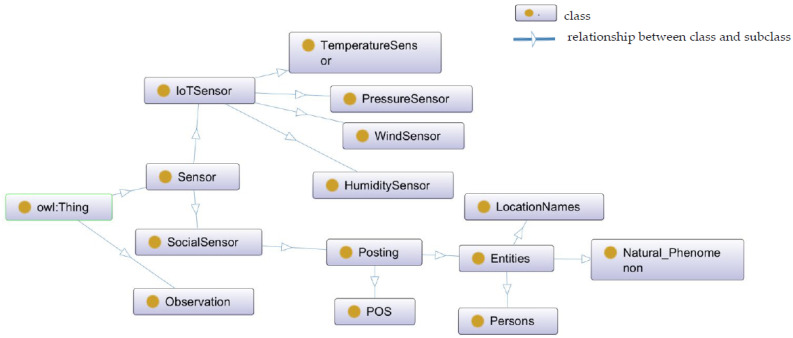
Top classes in semantic situation modelling.

The proposed situation model represents the classes, instances, rules, and their relationships when using combined information spaces. The top classes of the situation model are depicted in Figure 2. A detailed description of all the prefixes is presented in Table 2.

## 5. Mapping Sensor Data to a Semantic Situation Model

The “Semantic Sensor Network” (SSN) ontology is an ontology for describing sensors, their observations, observation procedures, and features of interest, etc., the W3C Semantic Sensor Network Incubator Group. SSN includes a self-contained core ontology called Sensor, Observation, Sample, and Actuator (SOSA) for its elementary classes and properties and contains a wide range of modules. Given our interest in describing the core concepts of the weather situation using combined information spaces, we focused on the following classes from the SSN ontology:

- sosa: Sensor to describe the sensors;
- sosa: Observation to describe the measurement context.

In addition to the class definition, we also used the main object properties associated with these classes: sosa: observedProperty, sosa: madeObservation, sosa: observes, sosa: hasResult, etc. These properties were used with the idea of forming a “subject–predicate–object” triple structure. For instance, the object property madeObservation connects the class Sensor and class Observation in “Sensor-madeObservatio-Observation”.The Ontology for Meteorological Sensors (AWS) [41] is based on the technical literature published by the World Meteorological Organization (WMO) (https://public.wmo.int/en) ontology which extends the SSN ontology and provides descriptions of different models of sensors used to measure weather phenomena. While AWS does not import any ontology, it proposes a wide variety of sensor models in the AWS Hierarchy. Given our interest in weather situation descriptions and with the data being sourced from the Bureau of Meteorology sensors, we did not try to specialize the sensors and only reused the sensor models closely related to the weather phenomena being observed. More specifically, we mostly reused the following classes:

- aws: AtmosphericPressureSensor for pressure measurements;
- aws: HumiditySensor for humidity measurements;
- aws: TemperatureSensor for temperature measurements;
- aws: WindSensor for wind measurements.

**Figure 3:**
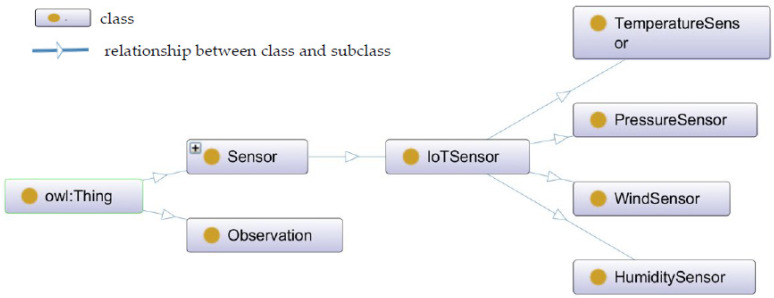
Top situation model classes for mapping IoT sensor data.

The AWS ontology relies on domain-specific definitions sourced from the Climate and Forecast Metadata Conventions, which includes a collection of standard names (http://www.met.reading.ac.uk/~jonathan/CF_metadata/14.1/#standard_names) for climatic data variables which was published by Jonathan Gregory and aims at providing a comprehensive and systematic description for climatic data variables. The Climate and Forecast features ontology is a translation of the Climate and Forecast (CF) standard names vocabulary and contains classes that specialize the sosa: FeatureOfInterest class. We leveraged this vocabulary to (a) define the weather features of interest such as temperature, pressure, humidity, and wind, etc., and (b) define the measurable properties such as air_temperature, air_pressure, air_relativehumidity, wind_direction, wind_speed, wind_gust for sosa: FeatureOfInterest. GeoSPARQL [42] is one of the most widely used vocabularies for describing geometries of spatial objects and it also extends one of the most common vocabularies in WGS84 which is used for representing spatial coordinates. This ontology can be used to define the vocabulary for representing geospatial data and to spatially link IoT sensor and social information spaces. The point geometry from GeoSPARQL can be used to describe the location of IoT sensors and social sensors. For describing the timestamps associated with IoT sensor observations and social media postings, the W3C Time ontology [43] can be used. This ontology enables the description of both time instants and intervals. The authors of [42] reused the classes time:Interval and time:Instant from the W3C Time ontology and the ISA Core Location vocabulary [44] to describe the experimental farm address. We used the sosa:resultTime object property to represent the instant of time an observation is made. The ontology for the proposed situation model described in Section 7 was created using Protégé. This situation model contained the minimum set of classes that can describe IoT sensors, their observations, social sensors, and their postings. The top classes and the main object properties of IoT sensors in the proposed situation model are shown in Figure 3 and Table 3 shows the object properties, domain, range, and the description of main object properties.

## 6. Mapping Social Media Data to a Semantic Situation Model

To describe the social media observations, we designed a social media ontology with the namespace senso: http://dai1.dg-001.cloud.edu.au/ontologies/senso to describe the postings made by a social sensor using the following classes:

- senso: Posting to describe the social media posting;
- senso: Entities to specify the entities to be extracted from social media postings.

**Figure 4:**
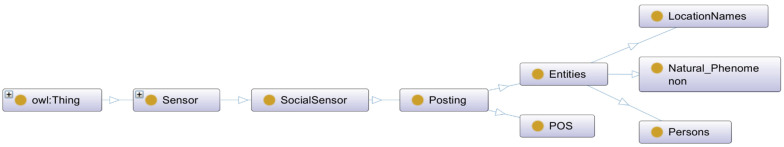
Top situation model classes for mapping social media postings.

The senso: Posting class was used to describe a set of concepts such as location or persons that are being discussed in the social media posting. These classes were intended to serve as a source of additional situational information to what can be achieved using IoT sensors only. Further, to the class definitions, we also defined object properties associated with these classes such as senso:isReportingOn, senso: hasPersonCount, senso: hasmentionedLocation, senso: hasCity, senso: similarityStrength, etc. These properties were used with the idea of forming a “subject–predicate–object” triple structure. For instance, the object property hasmentionedLocation connected the class Posting and class LocationNames in “Posting-mentionsEntities-LocationNames”, identifying a relation between a Posting and the locations that are mentioned in the posting. The hasPersonCount connected the class Posting and a value in “Posting-hasPersonCount-value” identifying a relation between a Posting and the value representing the number of persons mentioned in the posting. The ontology for the proposed situation model described in Section 7 was created with Protégé. The situation model contained classes that provide the descriptions of social sensors and their observation via social media postings. The object properties in an ontology describe relations between their respective domain and ranges. The top classes and the main object properties of social media postings in the proposed situation model are shown in Figure 4 and Table 4 shows the main object properties, their corresponding domain, range, and description.

## 7. Combining and Intersecting Social Media Data and Sensor Data into a Semantic Situation Model for Improved Situation Awareness

**Figure 5:**
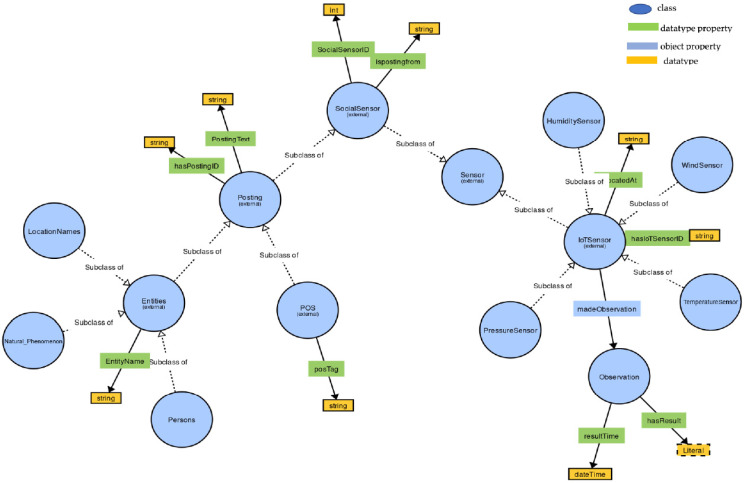
Classes, object, and data properties of a wind gust situation model in a combined information space.

In this paper, we model the wind gust-related situation. As we noted in Section 4, we did not aim to devise a comprehensive semantic situation modelling ontology from scratch, but we rather reused and combined concepts from existing ontologies that have been used to model sensor or social media information from the perspective of providing situation awareness by intersecting sensor and social media information spaces. For example, given our interest in describing the core concepts of the wind gust situation in the combined information space, we focused on the following classes: from the SSN ontology we used the classes sosa: Sensor to describe both IoT and social sensors sosa: Observation to describe the measurement context, the object properties associated with these classes: sosa: observedProperty, sosa: madeObservation, sosa: observes, sosa: hasResult, sosa: hasFeatureOfInterest, etc. The senso: Posting class, object properties such as senso:isReportingOn, senso: hasPersonCount, senso: hasmentionedLocation, senso: hasCity, senso: similarityStrength, etc., were reused from the social media space. In addition, we used the classes of the aws ontology to capture wind measurements from windSensor. As in a general ontology, the class hierarchy can be better analyzed using object and data properties. In our ontology, object properties represented the connection between a subject and an object via the predicate whereas the data properties represented the connection of a subject to a form of data attribute. A detailed description of all the object properties is presented in Table 3 and Table 4. In Figure 5, the classes, object, and their data properties of a wind gust situation model in a combined information space are shown, whereas the object properties, domain, range, and the description of main object properties in combined information space are presented in Table 5.

The real-time availability of social media data makes these data a valuable resource for situation awareness. The volume, velocity, structureless, heterogeneity, and enormous volumes make it challenging to process such data. Further, the volume and velocity of social media posts tend to be extremely high during times of an event, making the filtering of relevant situational data a complex and challenging task [36]. Moreover, filtering relevant situational data from social media data spaces is further complicated by the short, inconsistent nature of social media postings [16] and their high volumes make it time-consuming to filter relevant situational data [35]. The volume of data generated by social media varies based on the extent of emergency events as the no of affected people and geographical area vary [8] as does the adoption of social media [45] (number of active users) in the affected area.

While a significant amount of research has focused on retrieving huge volumes of data that may or may not provide a good reflection of the situation being understood and then processing the extracted information to understand situations, it is still a challenge to identify and filter relevant situational information. Situational data from social media are commonly filtered by querying social media data spaces using either keywords, hashtags, or geotags [19]. The process for mapping the social media data to the ontology is discussed in detail in Section 8.1. In this section we discuss how we designed rules that were used for identifying and extracting relevant social media postings within the spatial boundary of the IoT sensors. Overall, we followed the process of (a) filtering social media postings from the social media data stream using the Twitter academic research API, (b) processing social media data to remove noise, and (c) extracting high value information which includes temporal and spatial information and synonyms of IoT sensor concepts (e.g., temperature—hot) and information on the entities such as names of places (Melbourne, etc.) and persons (Dimitrios, etc.) mentioned in the postings and their count.

## 8. Evaluation of Intersecting the Information Spaces of Sensor Data and Social Media Postings

### 8.1. Test Bed Implementation for Evaluation

We designed and implemented a highly scalable, fault-tolerant architecture for facilitating the development of internet-scale IoT sensors and social media-based situation awareness systems. With this highly scalable architecture, we designed various layers that can efficiently identify, extract, store, process, and visualize data with a high volume, velocity, and variety. For data ingestion from various IoT sensors and social media platforms, we made of use of an Apache Kafka cluster and an Apache spark cluster for data processing. An Apache Jena-based cloud instance was used for storing the data into a highly scalable triple store. Apache Kafka is a fault-tolerant, highly scalable, and available open-source distributed streaming platform that can be used to store and process data streams. It primarily consists of topics, producers, and consumers. Topics are logical entities where data records are published by producers and consumers read data records from topics. We made use of various Kafka Producer applications to ingest data from various automatic weather stations from the Bureau of Meteorology in the state of Victoria and the geotagged social media postings from Twitter. Twitter is one of the most popular and widely used social media platforms. The Kafka producer application connects to the Twitter streaming API v2 via an academic endpoint and is capable of using either keywords, hashtags, geotags, or a combination of these to filter tweets and then publishes the tweets into Kafka topics. A detailed discussion and implementation of the cloud-based system architecture is out of the scope of this paper. This section describes the process that we followed for extracting IoT sensor data from various weather stations across the state of Victoria. Then we discuss the algorithm that translates the IoT sensor data into high value information and annotates the high value information based on the concepts in the IoT sensor (SOSA) ontology and converts it into triples as well as storing the triples to an Apache Jena Fuseki triple store. We also present a brief description of the Bureau of Meteorology weather stations and their sensors.

The Bureau of Meteorology is Australia’s national weather, climate, and water agency. Weather data are obtained from different automatic weather stations around Australia and the latest weather observations web pages are updated from the Bureau of Meteorology’s real-time database. Table 6 represents an extract of various sensors from the Melbourne (Olympic Park) weather station. The individual observations such as temperature, pressure, humidity, wind speed, direction, and gust describe the context of the respective sensor measurements and the properties such as sosa:observedProperty, sosa:hasFeatureOfInterest, sosa:madeBySensor, and sosa:hasSimpleResult link the respective observations with their corresponding observed properties, natural phenomena, sensors, and measurement values.

**Figure 6:**
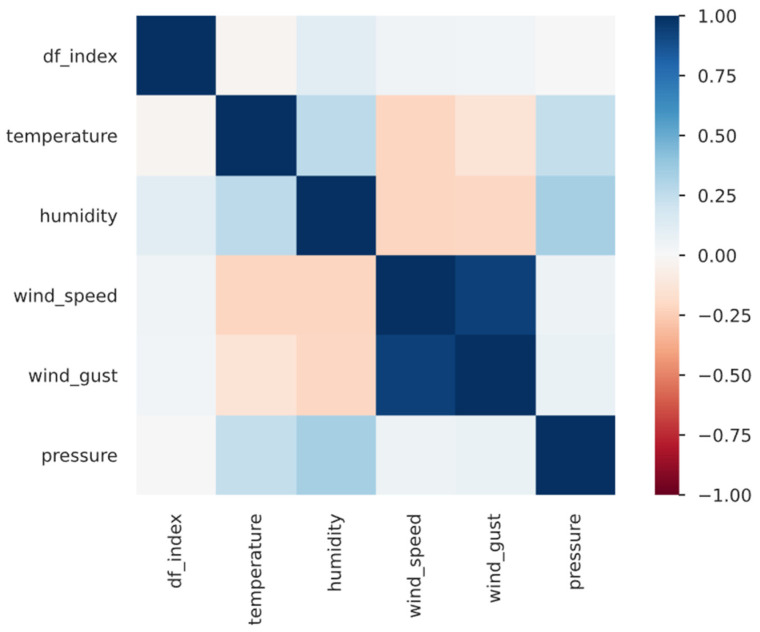
Correlation between weather features.

In this section we briefly describe the processes involved in RDF dataset generation. The measurements produced by the automatic weather station sensors were read at every 10-min interval and stored in a Kafka topic. It is possible that the data contained noise due to incorrect measurements. After reading the data we ensured the data were of decent quality by identifying inconsistent values such as ‘–’ and empty values. We also transformed the time stamp reported attached to the sensor measurements into a format suitable for time-based calculations. We also monitored the weather sensor data between 29 November 2021 and 9 February 2022 to identify any anomalies and correlations. Figure 6 shows the results from pearson correlation, applied to understand if there was any significant correlation between these measurements so that the situation model could be adjusted accordingly.

We developed an algorithm to clean and transform these measurements into an RDF format and was implemented in an OpenStack-based cloud instance. This algorithm leveraged an Apache Kafka cluster to poll the sensor data across the state of Victoria for every 10-min interval from Bureau of Meteorology web pages and stored it in Kafka topics. A copy of these measurements was also stored in a MySQL Database. The goal of this algorithm was to read and transform the sensor measurements for temperature, humidity, pressure, wind direction, speed, and gust based on the concepts described in the situation model and store them in the Apache Jena-based RDF triple store. In this section we briefly describe the working of the algorithm involved in RDF dataset generation. The first part of the RDF data generation involved cleaning the data from sensor observations and adding geographic information to this data. The second part involved transforming the cleaned data into an RDF format.

### 8.2. Sensor Data Cleaning

The weather data from the Bureau of Meteorology weather station is the raw form were mostly structured. As such the data did not require comprehensive cleaning and a sample of the raw sensor data is presented in Table 6. These data contained measurements from temperature, humidity, pressure, and wind sensors which are located in specific places such as Melbourne and each place has multiple weather stations that host these sensors. The measurements produced by the sensors from the weather stations were read at 10-min intervals between 29 November 2021 and 9 February 2022 and stored in a Kafka topic. It is possible that the data contained noise due to incorrect measurements. After reading the data we ensured that the data were of decent quality by identifying inconsistent values such as ‘–’ and empty values. These values were imputed with the respective mean values for each of the sensor observations. We also transformed the time stamp reported attached to the sensor measurements into a standard xsd:dateTime format as specified in the situation model. For example, the value of the timestamp reported in observations was 29 November 7:20 p.m. and it did not have a year component attached to it. We converted this to a standard format and added the year component as 21 November 2021 19:20:00 using the datetime library in python. These data were used in the next step for adding geographic information.

### 8.3. Adding Geographic Information

The observations at this point, however, did not have geographic information associated with them when they were being reported. The Bureau of Meteorology provides a list of all the weather stations with their precise geographic location. As presented in Table 7, each weather station is associated with basic details such as the official station name, a unique station ID, and geographical coordinates which help in identifying the weather station.

The next task was to map the geographic coordinates of the weather stations to cleaned data. We converted all the station names into lower case and created an index of the station names with their corresponding latitude and longitude values. We mapped this index with the station names from the cleaned data created in the sensor data cleaning step. The weather sensor data in the above-mentioned duration was also monitored to identify any anomalies and correlations. We applied the Pearson correlation to understand if there was any significant correlation between these measurements so that the situation model could be adjusted accordingly.

### 8.4. Sensor and Social Media Data Transformation

The transformation process, implemented in python, read the cleaned dataset, and produced a triple. The goal was to annotate the data based on the description provided in the situation model, and as such it was not feasible to use a generic library such as Rdflib to fulfill this requirement. We first created a dictionary using key value pairs to capture the descriptions of the sensors used, their location, and their units of measurement that are described by the situation model (Table 5). An example dictionary for wind gust description is show in Table 8.

To make it convenient to reference the ontologies during the transformation process, we the set the prefixes for each of the ontologies being used. For example, we set the prefix sosa for the ontology http://www.w3.org/ns/sosa/. A detailed description of all the prefixes is presented in Table 2. We then read each row from the dataset and matched the corresponding key value pairs in the dictionary to the values from each of the sensor. Consider a single record from the raw data as shown in the Table 9 below.

The corresponding transformed record is:

{‘unit’: ‘http://qudt.org/1.1/vocab/unit#MeterPerSecond’, ‘unit_txt’: ‘degreeAngle’, ‘unit_symbol’: ‘m/s’, ‘cdt_type’: ‘ucum’}

wind_gust

<http://www.w3.org/ns/sosa/sensor/86338> a sosa:Weather_Station ;

sosa:observedProperty <http://www.w3.org/ns/sosa/observableProperty/wind_gust> ;

sosa:hasFeatureOfInterest <http://www.w3.org/ns/sosa/FeatureOfInterest/wind> ;

sosa:madeBySensor <http://www.w3.org/ns/sosa/Sensor/windspeed_sensor> ;

geo:lat “−37.8255”;

geo:long “144.9816”;

senso:hasCity “melbourne” ;

senso:hasPlace “melbourne (olympic park)” ;

sosa:resultTime “2021-11-29 19:20:00”^^xsd:dateTime ;

sosa:hasSimpleResult “9 “^^cdt:.

The social media data for this study were gathered by leveraging the twitter-v2 academic research API from the Twitter platform. In this paper, we considered only geotagged tweets, and searched Twitter between the dates 29 November 2021 and 9 February 2022 to match the window of the IoT sensor observations from the Bureau of Meteorology. We extracted the specific point coordinates (latitude and longitude) for each of the weather stations in Victoria. We created a set of rules to search for tweets within twenty-five miles of each of these geo-coordinates. Then we filtered tweets which were not replies or quotes, were not retweeted and were only in the English language. We performed a basic sanitizing of social media postings to remove noise such as removing links from the tweets, but we left the hashtags as hashtags can still contain some information which can help us identify the conversations in a more general way. We then made the social media posting case insensitive to avoid the words such as ‘climate’ and ‘Climate’ being treated as different. We further tidied up the social media posting by removing the punctuation marks, double spacing, and Stopwords.

Consider an example social media posting. “Another couple of toasty hours on the bike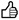 @ Nimmons Bridge https://t.co/K23JxdCBBU”. This posting was posted from Newtown in Australia. Our algorithm matched this tweet to its nearest weather station in Ballarat. This posting was found to be discussing temperature concept and one person was mentioned in the tweet. We verified this match by investigating the place name field returned by the tweet object. The field name for this specific posting contained a value of Newtown. Newtown is a locality situated on Pitfield Road (Lismore—Scarsdale Road) in Golden Plains Shire, about seventeen miles southwest of Ballarat.

Tweet country: Australia

Tweet place name: Newtown

Closest weather station: BALLARAT

Location mentioned: []

Observable property: temperature

Similarity score, 53.0

Person count: 1

<http://www.w3.org/ns/sosa/Sensor/1496441118602452997> a sosa:Sensor;

rdfs:label “Social_Sensor_from_BALLARAT”;

sosa:observes <http://www.w3.org/ns/sosa/observableProperty/temperature>;

sosa:madeObservation <http://www.w3.org/ns/sosa/Observation/temperature_observation_from_BALLARAT>;

sosa:resultTime “2022_02_23_11_05_39”^^xsd:dateTime;

senso:isReportingOn “temperature”;

senso:similarityStrength “53.0percent”;

senso:hasPersonCount “1”;

senso:hasmentionedLocation “[]”;

senso:hasCity “BALLARAT”.

As discussed in Algorithm 1, we developed a novel technique to estimate the similarity of a social media posting to an observable property. For example, specific keywords extracted from social media postings that were synonymous with sosa: ObservableProperty such as Temperature which is an Observable property of an IoT sensor. This technique makes use of natural language processing-based semantic similarity approaches to estimate how similar a social media posting is to the observable property described in the situation model. This estimation helped us determine the potential social media postings similar to the observable property using token similarity techniques from spacy. We first normalized the social media postings to remove all punctuation, weblinks, users such as @user information, hashtags, emojis, etc. We then performed tokenization and computed a semantic similarity estimate for each of the token in the social media posting and the observable property. Any token with a score equal to 1 or less than 0.5 was filtered out to avoid any ambiguous matches. We considered the maximum similarity score among the observable properties for each social media posting to estimate its closeness to the observable property based on the assumption that the higher the scalar similarity score the more similar the posting is to the observable property.
**Algorithm 1: Match social media posting with sosa:observable property**procedure GETSOSAOBSERVABLEPROPERTY(*socialMediaTokens*) *smTokens* ←nlp(*socialMediaTokens*) *sosaObservableProperties* ←*temperature,humidity,wind,gust,pressure* *observablePropertiesTokens* ← nlp(*sosaObservableProperties*) *tokenSimilarity* ← [] *dictTokenSimilarity* ← [] for *eachToken* in *observablePropertiesTokens* do for *eachsmToken* in *smTokens* do if *eachToken*.*text* != *eachsmToken*.*text* and *eachtoken*.similarity(*eachsmToken*) > 0.50 then  *tokenSimilarity*.append(*eachToken*.similarity(*eachsmToken*))  *dictTokenSimilarity*[*eachToken*.text]=*eachToken*.similarity(*eachsmToken*) end end if max(*dictTokenSimilarity*.items(), key ←lambda x : x[1]) is None then NSOP , 0 ; /* NSOP ← NoSimilarObservableProperty ∗ / *tokenSimilarity*.append(*eachToken*.similarity(*eachsmToken*)); *dictTokenSimilarity*[*eachToken*.text]=*eachToken*.similarity(*eachsmToken*) else *mostSimilarObservableProperty*,*mostSimilarObservablePropertyValue* =max(*dictTokenSimilarity*.items(),key ←lambda x:x[1]) ; /* return the most similar observable property(token) with max similarity of all tokens */ return *mostSimilarObservableProperty*, *mostSimilarObservablePropertyValue* end end end procedure

To identify and extract the concepts such as persons, geographical entities, and natural phenomenon, described in the situation model, we developed an algorithm that (a) reused named entity recognition techniques from spacy to support this extraction and (b) identified person names by applying parts of speech tagging. In the literature, named entity recognition techniques have commonly been used to identify entities mentioned in the social media postings such as geographical entities and natural phenomenon. To identify person names, we first applied parts of speech tagging to find out the parts of speech of each token in the tweet and looked for pos tag type ‘NNS’ (plural nouns) and created chunks of pos tags. Then for each of for each of these chunks we processed them individually till we found a chunk which is of tree type and contains the entity data. We then expanded the tree into leaves and attached the leaf names to the persons concept described in the situation model. Following the extraction of high value information from social media postings based on the concepts from the situation model using the techniques discussed above, we then translated and stored this information as triples to an Apache Jena Fuseki triple store as discussed in Section 8.4.

### 8.5. Evaluation of the Intersection of Social Media and Sensor Information Space

We tested the response times to validate the performance of the querying engine in terms of query response time by querying the sensor, social media, and combined information space with various queries. To count all the triples in database, the query response time was 4.535 s and the query returned 71,208 triples. Of these triples, it took 0.038 s to find out how many types of weather sensors were currently reporting observations and to retrieve the sensor name and the name of weather station as of 29 November 2021 19:30:00. When we searched for all sensors monitoring a specific location with coordinates (144.9816, −37.8255), the query took about 0.035 s and 0.175 s to extract all observations of a wind gust sensor.

To demonstrate the potential of using both IoT sensor and social media information spaces for improving situation awareness, we began by querying the IoT sensor and social media information spaces for various weather-related situations. More specifically, we considered the wind-related weather situation for evaluation in this paper. As seen in Table 10, there was an instance of a social sensor discussion at close to 5 a.m., related to gust near Puckapunyal West (defence) with a strength of about 72% and count of 1 person was mentioned. According to the Bureau of Meteorology, wind gusts can be 40% stronger than the forecasted average wind speed which means the wind gusts are normally 40% higher than the average. Based on this calculation, Table 11 (adapted from [46]) shows the potential gust ranges for their corresponding average wind speeds ranges. In the social media information space, we looked for any discussions on gust-related discussion and whether this discussion mentioned any locations and were any people observed in the discussion.

To look for more information, we then performed a union of sensor and social media information space to see how the gust measurements looked in the sensor information space from the Puckapunyal West (defence) weather station. To perform this search, we created a window of 1 h and took the average of the gust measurements in this hour. If it was the first measurement of the day from this weather station, then we expanded the window to 3 h. The measurement from Puckapunyal West (defence) for wind direction., wind speed, and wind gust returned values of SSE, 28 and 39, respectively, at 6 a.m. The social sensor reported that one person was involved in this gust situation in Puckapunyal West (defence) which corresponded to coordinates (−37.0177, 144.8546) and complemented the gust strength of 39 from the sensor which corresponded to a strong weather warning. By intersecting this with the social media information space, we were able to find additional information in terms of the number of people involved in this situation.

### 8.6. Evaluation Results

**Figure 7:**
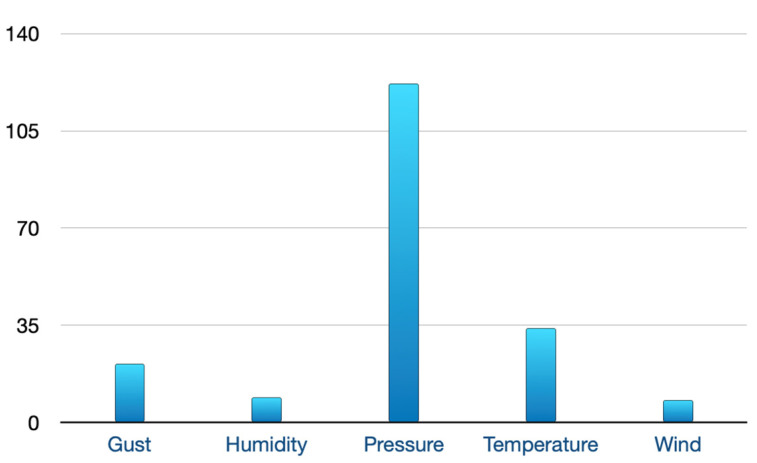
Social media postings matching the sosa observable property.

The query processing times were low when using either sensor or social media information spaces as well as combined information spaces. During the extraction of social media postings, we used bounding box coordinates to map the tweet to the nearest weather station by identifying if the weather station location fell within the bounding box attached to the social media posting. Our algorithm was able to match most of the social media postings to the correct weather stations. This could also be confirmed by looking at the place name information returned by the twitter with every posting. In twitter-v2 API, Tweets with a Twitter “Place” contain a polygon, made up of bounding box coordinates which normally refer to the place from which the user is posting. This field commonly corresponds to a name where the place is located within a country. Consider the example social media posting discussed in Section 8.4, this posting was posted from Newtown in Australia. Our algorithm matched this posting to its nearest weather station in Ballarat. This posting was found to be discussing the temperature concept and one person was mentioned in the posting. We verified this match by investigating the place name field returned by the tweet object. The field name for this specific posting contained a value of Newtown. Newtown is a locality situated on Pitfield Road (Lismore—Scarsdale Road) in Golden Plains Shire, about seventeen miles southwest of Ballarat. The social media postings dataset contained a total of 862 tweets, a majority (668) were returned as having a similarity score of 0.0. from the remaining postings, Figure 7 shows a distribution of the social postings that matched the sosa observable property from the situation model.

However, there were few false positives which were incorrectly matched to the observable property. For example: “Bairnsdale Bearings is an agent for Schmalz vacuum technology. If you have a worksite that has repetitive or manual handling, we can design a system for you that will save time and injuries” was related to an observable property “pressure”. We hope to improve the algorithm in future work to exclude such cases.

When we queried the combined information spaces, the volume of results returned by the query was quite low. This could be because when filtering the social media postings from Twitter, we considered using only geotagged tweets which commonly have a low volume. For future work, we would consider including all tweets within a given time period and move deeper into the places being discussed in the posting and extract the location information from these postings while also adding other information such as the infrastructure involved in the social media posting discussion.

## 9. Conclusions and Future Research

The development of situation models for integrating sparse IoT sensor data and social media postings, as well as techniques for fusing such information has the potential to provide richer and more accurate situation awareness information. In Section 8 we explained how we can transform the sensor and social media data to the situation model concepts that can be used to look for more information. However, with only limited public data available, opportunities to combine sensor and social media information data were low. In this paper, we proposed a situation model and various techniques for mapping IoT sensor and social media data to the situation model. In our future research, we aim to investigate a deeper extraction of high value information from social media, for example, distinguishing singular and plural nouns for persons and expanding the situation model to provide a more comprehensive description of situations for improving situation awareness by intersecting IoT sensor and social media information spaces. We also aim to develop tools for easy creation of situation models via picking and relating concepts for existing ontologies used to describe sensor and social media information spaces.

## Figures and Tables

**Table 1:** Situations, concepts, features of interest, and ontology in the IoT sensor information space.

Author	Situation	Concepts	IoT Sensor Information Space Features	Ontology
[28]	Deducing different activities of a car	Taxicab	Observation Record ID, Data Timestamp, Area ID, Location (longitude and latitude), Velocity, Driving direction, Taxi ID	High level static OWL Ontology
[27]	Early detection of a vehicle collision, health issues with drivers, and accidents involving dangerous goods	Driver, Vehicle	Driver’s ECG, Heart Rate, Accelerometer Position, Speed	SAREF, LogiCO
[29]	Increase volume setting in users’ cell phone, depict sleeping status in an airport lounge sleeping facility	Location, Person, Activity	Temperature, Latitude, Humidity Level, Longitude	SIoT
[30]	smart spaces	Car, smart home, workplace, hotel	Temperature, Humidity, Occupancy	VO/CVO
[32]	Controlling home appliances using natural language	State, Sensor, Action, Alert, Service	StartAction, StopAction, OffState, PausedState, Water Level, Temperature, Coffee Level	Home Appliances
[31]	Network Security	Context, Attack, Vulnerability, and Network Flow	DestIp, DestPort, Protocol, SourceIp, ICMPtype, ICMPcode	Network Security

**Table 2:** Ontologies that can be used to model situations in the proposed situation model when using combined information spaces.

Prefix	Ontology	Description
sosa	http://www.w3.org/ns/sosa/	Sensor, Observation, Sample, and Actuator (SOSA) ontology provides a lightweight core for SSN and aims at broadening the target audience and application areas that can make use of Semantic Web ontologies
ssn	http://www.w3.org/ns/ssn/	SSN ontology describes sensors and their observations and does not describe time, locations, etc.
senso	http://dai1.dg-001.cloud.edu.au/ontologies/SenSo	Senso ontology describes social sensors and their postings
aws	https://www.w3.org/2005/Incubator/ssn/ssnx/meteo/aws	AWS ontology provides descriptions of various sensor models used for measuring weather phenomena
time	https://www.w3.org/2006/time#	This ontology provides a vocabulary for expressing facts about topological (ordering) relations among instants and intervals, together with information about durations, and about temporal position including date-time information.
ns1	<http://www.w3.org/2003/01/geo/wgs84_pos#>	An RDF/OWL vocabulary for representing spatial information.
xsd	https://www.w3.org/2001/XMLSchema#	XML Schema namespace as defined by XSD
rdf	http://www.w3.org/1999/02/22-rdf-syntax-ns#	“RDF Schema for the RDF vocabulary terms in the RDF Namespace” (“World Wide Web Consortium (W3C)”)
rdfs	http://www.w3.org/2000/01/rdf-schema#	RDF Schema is an extension of RDF vocabulary, providing a data-modelling vocabulary for RDF data
owl	http://www.w3.org/2002/07/owl#	“This ontology partially describes the built-in classes and properties that together form the basis of the RDF/XML syntax of OWL 2.” (“World Wide Web Consortium (W3C)”)

**Table 3:** The object properties, domain, range, and the description of main object properties.

Object Property	Domain	Range	Description
sosa: madeObservation	sosa: Sensor	sosa: Observation	Relation between a Sensor and an Observation made by the Sensor
sosa:resultTime	sosa:Observation	xsd:dateTime	The instant of time when the Observation activity was completed
sosa:observes	sosa:Sensor	sosa:ObservableProperty	Relation between a Sensor and an ObservableProperty; capable of sensing
sosa:observedProperty	sosa:Observation	sosa:ObservableProperty	Relation linking an Observation to the ObservableProperty that was observed.
sosa:hasSimpleResult	sosa:Observation	value	The simple value of an Observation

**Table 4:** The main object properties, corresponding domain, range, and the description.

Object Property	Domain	Range	Description
senso:hasmentionedLocation	senso:Posting	senso:LocationNames	relation between a Posting and the location names mentioned in the posting
senso:similarityStrength	senso:Posting	value	relation between a Posting and the observable property which contains a value identifying the closeness of the observable property that is being discussed to the social media posting
senso:hasCity	senso:Posting	value	relation between a Posting and the name of the place it is posted from
senso: isReportingOn	senso:Posting	sosa:ObservableProperty	Relation between posting that are similar with ObservableProperty observed by social sensor
senso: hasPersonCount	senso:Posting	value	relation between a Posting and person, which contains a value representing the number of persons mentioned in the posting

**Table 5:** The object properties, domain, range, and the description of main object properties in combined information space.

Object Property	Domain	Range	Description
sosa: madeObservation	sosa: Sensor	sosa: Observation	Relation between a IoTSensor and an Observation made by the Sensor which is either an IoT sensor or social sensor.
sosa:resultTime	sosa:Observation	xsd:dateTime	The result time is the instant of time when the Observation activity was completed by either an IoT sensor or social sensor.
sosa:observes	sosa:Sensor	sosa:ObservableProperty	Relation between a Sensor (either IoT or social media) and an ObservableProperty that it is capable of sensing.
sosa:observedProperty	sosa:Observation	sosa:ObservableProperty	Relation linking an Observation to the ObservableProperty that was observed.
sosa:hasSimpleResult	sosa:Observation	value	The simple value of an Observation.
senso:hasmentionedLocation	sosa: Sensor	senso:LocationNames	relation between a social media sensor and the location names mentioned in the posting.
senso:similarityStrength	sosa: Sensor	value	Relation between a social media sensor’s posting and the observable property which contains a value identifying the similarity of the observable property that is being discussed in the social media posting.
senso:hasCity	sosa: Sensor	value	Relation between social sensor and the name of the place the social sensor or IoT sensor is reporting from.
senso: isReportingOn	sosa: Sensor	sosa:ObservableProperty	Relation between social media sensor and the sosa observable property that is being reported by social sensor.
senso: hasPersonCount	sosa: Sensor	value	Relation between a social media sensor and number of persons mentioned in the social media posting.

**Table 6:** Weather observations from automatic weather stations in Victoria.

Place_Name	Station_Name	Wind_Direction	Wind_Speed	Wind_Gust	Time_Reported
melbourne	Melbourne (Olympic Park)	SSW	6	9	29 November 7:20 p.m.
ballarat	redesdale	ENE	11	15	23 February 2022 18:10:00
bendigo	kyabram	ENE	11	17	23 February 2022 18:00:00
geelong	ferny creek	ESE	11	15	23 February 2022 18:10:00

**Table 7:** Weather station details in the state of Victoria.

Station ID	Station Name	Latitude	Longitude
86338	Melbourne (olympic park)	−37.8255	144.9816
83084	Falls creek	−36.8708	147.2755
87031	Laverton	−37.8565	144.7565

**Table 8:** Dictionary for wind gust descriptions.

Sensor	Location	Units of Measurement
**‘**wind_gust’: {‘observedProperty’: ‘wind_gust’, ‘madeBySensor’: ‘windspeed_sensor’, ‘featureOfInterest’: ‘wind’, ‘resultTime’: ‘instant’, }	{ ‘place_name’: {‘place_name’: ‘City’, }, ‘station_name’: {‘station_name’: ‘Place’, }, }	‘wind_gust’: {‘unit’: ‘http://qudt.org/1.1/vocab/unit#MeterPerSecond’, ‘unit_txt’: ‘degreeAngle’, ‘unit_symbol’: ‘m/s’, ‘cdt_type’: ‘ucum’, }

**Table 9:** Sample observation from the Melbourne Olympic Park weather station.

Attribute	Value
place_name	melbourne
station_name	melbourne (olympic park)
temperature	23.2
humidity	56
wind_direction	SSW
wind_speed	6
wind_gust	9
pressure	1014.3
time_reported	29/11/2021 19:20
lat	−37.8255
long	144.9816
station_id	86338

**Table 10:** Instances of social sensor discussions on gusts.

Socensorname	Strength	City	Count_People
Social_Sensor_from_PUCKAPUNYAL_WEST_(DEFENCE)	72.0%	Puckapunyal West (defence)	1

**Table 11:** Wind speeds, gusts, and associated wind warning categories.

Average Wind Speed (Knots)	Gust Strength (Knots)	Wind Warning Thresholds
10	14	
15	21	
20	28	
26–33	36–45	strong wind warning issued
34–47	48–65	gale force warning issued
48–63	67–88	storm force warning issued
64 or more	90 or more	hurricane force warning issued

## Data Availability

Not applicable.
